# Clinico-Pathological Spectrum of Alveolar Soft Part Sarcoma: Case Series from a Tertiary Care Cancer Referral Centre in India with a Focus on Unusual Clinical and Histological Features

**DOI:** 10.5146/tjpath.2023.01605

**Published:** 2024-05-18

**Authors:** Kanwalpreet Kaur, Amisha Gami, Ashini Shah, Jahnvi Gandhi, Priti Trivedi

**Affiliations:** Department of Oncopathology, Gujarat Cancer and Research Institute, Ahmedabad, India

**Keywords:** Alveolar soft part sarcoma, IHC, TFE3, Apple bite nuclei

## Abstract

***Objective: *** Alveolar soft part sarcoma (ASPS) is characterized by distinctive histomorphology of variably discohesive epithelioid cells arranged in nests and translocation of t(x;17) (p11.2;q25) resulting in *ASPSCR1-TFE3* fusion. The aim of the present study is to review the clinical, histopathological, and immunohistochemical profile of ASPS with a focus on unusual histological features.

***Material and Methods:*** The present study is retrospective and descriptive. All cases with a diagnosis of ASPS were retrieved with clinical and radiology details.

***Results: *** 22 patients of ASPS were identified. The most common site was the lower extremity and the size range was 3-22 cm. 54.5% of the patients had metastasis, with the lung as the most common site. Metastasis preceded detection of primary tumour in two cases. All cases showed similar histopathology of monomorphic epithelioid cells arranged in nests encircled by sinusoidal vasculature. Architecturally, the organoid pattern (81.8%) was followed by the alveolar pattern. 68.2% of the cases showed apple bite nuclei as the predominant nuclear feature. Rare nuclear features included binucleation (n=13), multinucleation (n=8), pleomorphism (n=4), nuclear grooves in three cases and intranuclear inclusion in one case, mitosis (n=5), and focal necrosis (n=6). All cases were positive for TFE3 and negative for AE1/AE3, EMA, HMB45, PAX8, MyoD1, SMA, synaptophysin, and chromogranin. Only two cases showed focal S100 positivity while one showed focal desmin positivity.

***Conclusion:*** Diffuse strong nuclear TFE3 positivity is sensitive for ASPS in an appropriate clinicoradiological context. Due to the high propensity for early metastasis, complete metastatic work-up and long term follow up is recommended.

## INTRODUCTION

Alveolar soft part sarcomas (ASPS) are rare soft tissue tumors of uncertain histogenesis having a distinctive histomorphological appearance of variably discohesive epithelioid cells arranged in nests and have a specific translocation of t(x::17)(p11.2;q25) resulting in *ASPSCR1-TFE3* fusion (1).

Marked histologic overlap with other tumors, and tumor at unusual site and unusual clinical presentation with mass at the metastatic site prior to the identification of the primary make the diagnosis tricky. The differential diagnoses include a broad range of mesenchymal and non-mesenchymal neoplasms such as paraganglioma, PEComa, granular cell tumor, metastatic carcinoma such as metastatic renal cell carcinoma, hepatocellular carcinoma, and adrenal cortical carcinoma.

The present study analyzes the clinical, histopathological, and immunohistochemical profile of ASPS and clinical outcomes in cases, wherever available. Particular emphasis was given on the unusual histological features. The differential diagnosis and potential pitfalls in the current era of the increasing spectrum of TFE3 rearranged tumors have been highlighted.

## MATERIAL and METHODS

The present study is retrospective and all cases with a histopathological diagnosis of ASPS were retrieved from the archives of the department of Oncopathology from 2012 to 2021 at a tertiary care cancer center. Demographic, clinical, and radiological data were retrieved from the case records. Cases with non-availability of either immunohistochemistry (IHC) or paraffin blocks were excluded. Histomorphological and immunohistochemical characteristics were analyzed in each case. TFE3 immunohistochemistry was performed wherever unavailable. The histological parameters evaluated were growth pattern, presence of crystals confirmed by periodic acid-Schiff stain with diastase (PAS-D), nuclear features, presence of inflammation, fibrous septa, vascular invasion, necrosis, cystic change and myxoid change.

## RESULTS

A total of 22 patients (0.4%) with ASPS out of 5541 soft tissue sarcomas were identified from 2012 to 2021. The patient age range was 2-47 years and the median age was 27 years. The M:F ratio was 0.8:1. The most common site was the lower extremity in 45% (10/22) of the cases followed by the upper extremity 27.3% (6/22) of the cases, retroperitoneum in 18.1% (4/22) cases and one case each in the head and neck, chest wall, and lung. Clinical, radiological, and outcome details are given in Table 1.

**Table 1:** Clinico-radiological characteristics

**S. no**	**Age** **Sex**	**Site**	**Size**	**Radiology**	**Mets** **M/S**	**AJCC** **TNM**	**Treatment**	**Follow up and outcome**
**1**	29F	Left gluteal mass	9 cm	Lytic sclerotic lesion in left ilium with large irregular extrapelvic vascular soft tissue component p/o primary bone sarcoma	Lung (S)	T2N0M1 Stage IV	Doxorubicin+ Cyclophosphamide+ Vincristine+ Mesna x 8 cycles +RT	12 months: No response Tumor size increased to 13 cm + lung and brain dural based metastasis
**2**	3F	Post auricular	3 cm	Heterogenous soft tissue lesion noted involving right retroauricular region	Lung (S)	T2N0M1 Stage IV	Cyclophosphamide+ Dactinomycin+ Vincristine x 7 cycles	5 months: No response Tumor size increased to 3.7 cm
**3**	21F	Thigh	12.8 cm	Large lobulated T2 hyperintense soft tissue mass in the right vastus lateralis muscle. Multiple intralesional and prominent vessels seen in the lesion	Lung (S)	T3N0M1 Stage IV	Doxorubicin + cyclophosphamide+ mesna x 3 cycle àRT àgemcitabine and docetaxel	11 months No response Tumor size increased to 15.6 cm
**4**	24F	Chest wall mass	7.5 cm	Well defined heterogeneous soft tissue lesion noted in fascial plane between left pectoralis major and minor muscle, surrounded by tortuous dilated vessels	No	T2NxM0 Stage IIIA	WLE left chest wall with clear margins	5 months No evidence of disease
**5**	25M	Left Lung Upper lobe	11 cm	Heterogenous enhancing soft tissue density lesion with internal necrosis and foci of calcification in anterior segment of left upper lobe.	Liver Bone (M-1 year)	Stage 1a	RT+ doxorubicin and zoledronic acid x 4 cycles	12 months No response Tumor size increased to 11.9 cm with metachronous disseminated metastasis
**6**	21M	Iliac fossa	22 cm	Heterogeneously enhancing mass in right paramedian lumbar iliac fossa region with internal cavitation and necrosis	Posterior Fossa, lung and lymph node (S); liver and lung (M-1 year)	T4N1M1 Stage IV	Right suboccipital craniotomy and total excision of SOL + CT	24 months No response 22 cm tumor noted in abdomen extending to pelvis Liver + lung metastasis
**7**	2F	Forearm	4 cm	Altered signal intensity lesion Hypointense on T1W and hyperintense on T2W	No	T1NxM0 Stage I	Surgical resection with free margins	4 months No evidence of disease
**8**	40M	Left thigh	8 cm	Primary site radiology: NA	Lung (S) Lung and iliac bone mets (M-2 years)	T2N0M1 Stage IV	Doxorubicin+ gemcitabine x 9cycles	48 months No response Number and size of lung metastases increased from 1.3 cm to 5.4 cm
**9**	30F	Right thigh	13.5 cm	Clinical diagnosis: ca lung with mets in bone and brain	Bone Lung Brain (left occipital lobe) (S)	T3N0M1 Stage IV	Lost to follow up	
**10**	20M	Right Paraspinal region	9.3 cm	Heterogeneously enhancing lesion with internal cystic and necrotic areas, hypointense on T1and hyperintense on T2W images	No	T2N0M0 Stage IIIA	Laminectomy (incomplete resection) followed by palliative RT 30 Gy 10 cycles	12 months
**11**	22F	Abdominal wall in rectus	8 cm	**-**	No	T2N0M0 Stage IIIA	Wide local excision With clear margins	4 months No evidence of disease
**12**	30F	Right gluteal Region cystic swelling	10 cm	Large well defined multiloculated fluid collection with thick peripheral wall is seen in skin and subcutis with intramuscular extension to gluteal muscles	No	T2N0M0 Stage IIIA	Wide local excision With clear margins	72 months No evidence of disease
**13**	47M	Left forearm	10 cm	Primary site radio: NA CT head and neck: 7.5x5.2x5.0 cm3 heterogeneously enhancing soft tissue lesion involving bilateral nasal cavities with internal necrotic areas, erosion of maxillary sinus, extension to the sphenoid and ethmoid sinuses	Axillary Lymph node Nasal cavity, Lytic mets in Skull (S- 3 months)	T2N1M1 Stage IV	WLE+ RT+ doxorubicin x 2 cycles	7 months No response Metastasis in nasal cavity
**14**	18M	Right calf	7 cm	NA	No	T2N0M0 Stage IIIA	WLE+ Doxorubicin+ RT	4 month
**15**	40M	Arm	3 cm	NA	Liver Bone (S)	T1N0M1 Stage IV	WLE+ Cisplatin+ doxorubicin x 9 cycles	108 months No evidence of disease
**16**	24F	Thigh	7 cm	NA	Lung (S)	Stage IV	WLE+ Doxetaxel+ Gemcitabine x 8 cycles+ RT	36 months No response Increase in size of lung metastasis from 1.9 to 2.5 cm
**17**	18M	Retroperitoneum	5 cm	Circumscribed heterogeneously enhancing soft tissue density lesion with internal necrosis in left lumbar region	No	Stage II	WLE+ Doxorubicin x5cycles	24 months No evidence of disease
**18**	3F	Left forearm	3.9 cm	NA	No	T1N0M0 Stage II	WLE	24 months No evidence of disease
**19**	28M	Right thigh	9.5 cm	Large lobulated altered signal intensity lesion involving anterolateral part of right upper thigh, iso to hypointense on T1W and heterogeneously hyperintense on T2W images	Lung Brain (S)	T2N0M1 Stage IV	RT 30 Gray 10 cycles + CT	96 months; Follow up radiological data NA
**20**	18 F	Left forearm	1.5 cm	NA	No	T1N0M0 Stage I	WLE	4 month, No evidence of disease
**21**	6M	K/C/O T-ALL, Paravertebral location on the left side after 2 years	3.0 cm	Heterogeneously enhancing soft tissue lesion in paravertebral location on the left side	Bone (S)	Stage IV	Laminectomy	24 months, No response
**22**	22F	Left thigh	2.5 cm	NA	Brain Lung (S)+ Scalp, Breast mets (M)	Stage IV	WLE+ RT	12 months

**NA:** Not Available; **S:** Synchronous Metastasis, **M:** Metachronous Metastasis

**Figure 1:**
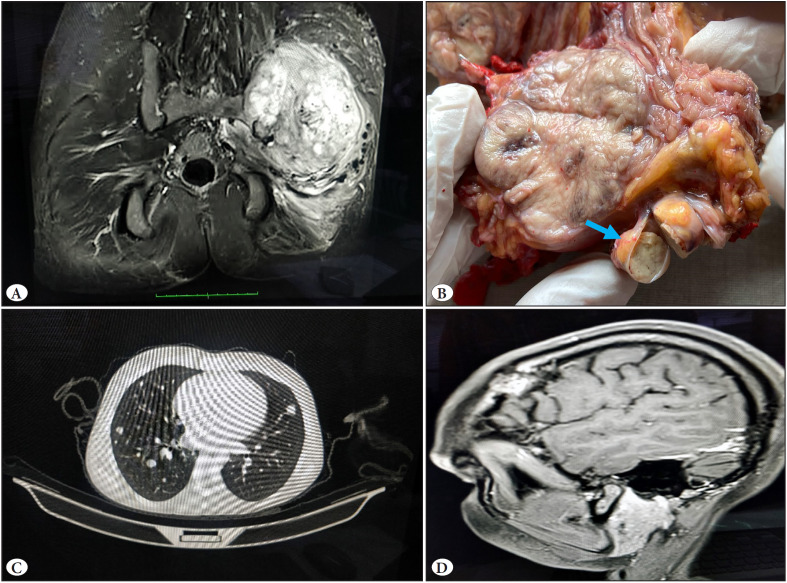
**A)** MRI pelvis showing heterogeneously enhancing 13x10x11 cm3 soft tissue lesion with lytic lesion in left iliac bone and left acetabulum which is hypointense in T1W and heterogenous hyperintense T2W, **B)** Gross of ASPS showing multilobular fleshy tumour with lymphovascular invasion, **C)** Axial CT thorax revealed presence of multiple metastatic lesions of variable sizes, diffusely scattered in both lung fields, **D)** CT skull show dural based metastasis.

Tumor size varied from 3 cm to 22 cm with a mean size of 7.8 cm. Lymph node metastasis was seen in 2 cases only. Distant metastasis (54.5%;12/22) was more frequent than lymph node metastasis. Out of these 12 cases with metastasis, 91.7% of the patients had synchronous metastasis while three showed metachronous metastasis. The lung was the most common site (90.9%) followed by the brain, bone, and liver (Figure 1). In one case of ASPS of the forearm, an unusual site of metastasis was bilateral nasal cavities, with biopsy showing a submucosal tumor. Five patients amongst these had multiple site metastasis. The metastasis preceded detection of the primary tumor in two cases. One case presented with a posterior fossa mass and the second case with a pathological fracture of the right femur, and both were diagnosed as ASPS on biopsy. Subsequently, PET revealed a primary mass in the left iliac region and the right thigh respectively. Thus, most cases presented with AJCC stage IV at the time of diagnosis (54.5%;12/22), followed by stage IIIa (22.7%; 5/22), stage I (13.6%; 3/22), and stage II (0.9%; 2/22).

One patient with T-ALL post remission showed ASPS in the left paravertebral location, post remission. The sibling of the patient also had T-ALL and developed glioblastoma 4 years post remission. The patient was further evaluated and diagnosed with constitutional mismatch repair deficiency syndrome (CMMRD) with a homozygous deletion (chr7:6026910; delC) detected in exon 11 of the *PMS2 *gene.

**Figure 2:**
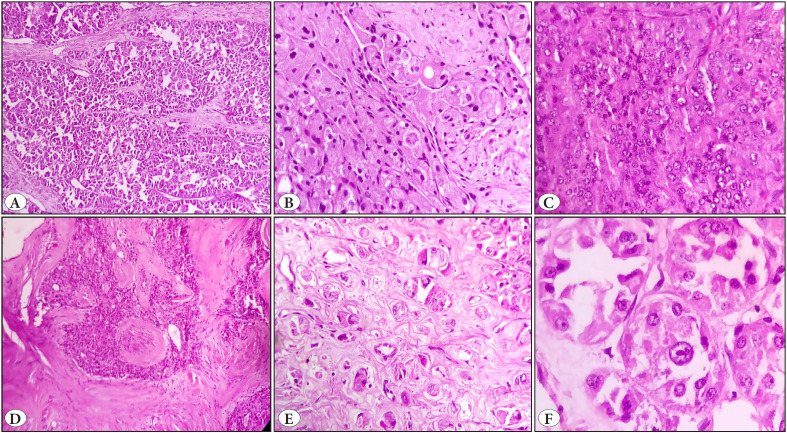
Histomorphological features of ASPS, **A)** Alveolar pattern (H&E, x100), **B)** Organoid pattern with spindling (H&E, x100), **C)** Solid pattern (H&E, x100), **D)** Thick fibrous septae (H&E, x100), **E)** Single cell infiltration in septae (H&E, x100), **F)** Classical vesicular nucleus with prominent eosinophilic nucleoli (H&E, x400).

Microscopically, all cases showed a multilobular architecture separated by fibrotic bands. The most predominant architecture pattern of the tumor cells within the lobules was the organoid pattern (81.8%;18/22) followed by the alveolar pattern (n=4) encircled by sinusoidal capillary vasculature (Figure 2). The size of the nests was variable with the number of cells in one nest varying from 10 cells to as many as 200 cells. Focal solid areas without any intervening vasculature were seen in 3 cases (Figure 2). Thick fibrotic bands were seen in 50% (n=11) of the cases (Figure 2). The rare architectural features noted were infiltration of single cells in septa and focal spindling of tumor cells in 3 cases each (Figure 2).

**Figure 3:**
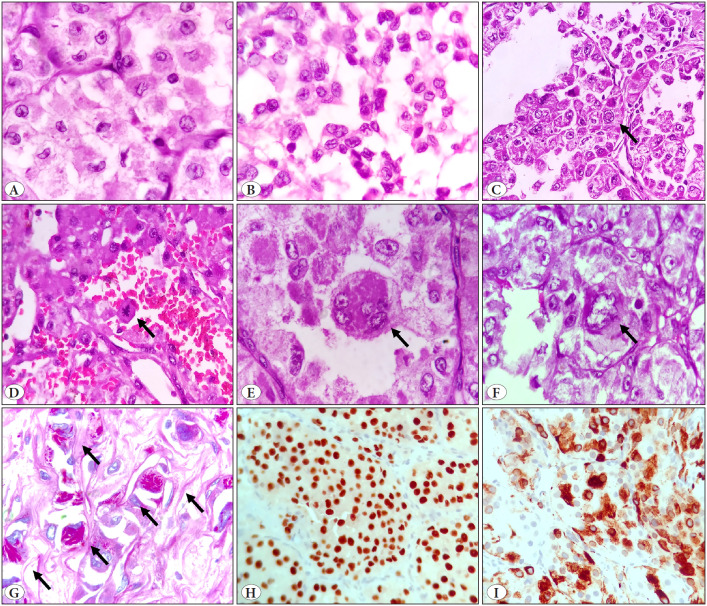
Unusual Histomorphological features of ASPS, **A)** crenated, crescent shaped apple bite nuclei (H&E, x400), **B)** nuclear grooves (H&E, x400), **C)** intranuclear inclusion (H&E, x400), **D)** mitosis (H&E, x400), **E)** multinucleation (H&E, x400), **F)** nuclear pleomorphism (H&E, x400), **G)** PAS D positive needle shaped crystals (PAS-D, x400), **H)** Diffuse nuclear TFE positive (IHC, x400), **I)** Focal desmin positive (IHC, x400).

Cytologically, tumor cells were epithelioid or polygonal with abundant eosinophilic granular cytoplasm in 91% of the cases and predominantly clear cytoplasm in 2 cases. It was also noted that the cytoplasm was more condensed near the nucleus and clearing towards the edge of the cell. The classically described round to oval nuclei with vesicular chromatin and prominent eosinophilic nucleoli with anisonucleosis was a major feature (>50% of the tumor nuclei) in only 31.8 % (7/22) of the cases (Figure 2) while in 68.2% (15/22) of the cases the majority of the nuclei showed wrinkling and a concave nuclear contour without nucleoli, described as apple bite nuclei (Figure 3). Rare nuclear features included binucleation (n=13), multinucleation (n=8), pleomorphism (n=4), and nuclear grooves in three cases and intranuclear inclusion in one case (Figure 3). Mitotic activity in general in ASPS is rare with only 5 cases showing occasional mitoses. Necrosis was infrequent and focally seen only in 6 cases. A lymphovascular embolus was a common phenomenon seen in 50% of the cases. None of our cases showed perineural invasion. Intratumoral hemorrhage in the center of the nests was seen in 2 cases.

Many cells with PAS-D positive rod-like crystalline structures in a sheaf-like or stacked configuration in the cytoplasm were seen in 5 cases while this was seen in occasional cells in 3 cases (Figure 3). There was no significant inflammatory host response in any case. There were focal intratumoral lymphocytes in 2 cases but peritumoral lymphocytes were seen in only one case. Other inflammatory cells such as plasma cells, granulocytes and histiocytes were absent.

IHC was performed in all cases to rule out paraganglioma, PEComa, granular cell tumor, metastatic carcinoma such as renal cell carcinoma, hepatocellular carcinoma, or adrenocortical carcinoma that can mimic ASPS as per the clinical context and morphological features. All cases showed diffuse nuclear positivity for TFE3 (Figure 3) and consistent negativity for AE1/AE3, EMA, vimentin, HMB45, PAX8, MyoD1, SMA, synaptophysin, and chromogranin. Only two cases showed focal S100 positivity while one showed focal desmin positivity (Figure 3). Histomorphological and IHC details are given in Table 2.

**Table 2:** Histopathological and immunohistochemistry features.

**S .no**	**Organoid** **pattern**	**Alveolar** **pattern**	**Solid** **pattern**	**Fibrosis**	**Vesicular Nuclei with prominent nucleoli**	**Concave,** **Crescent,** **Apple bite** **nuclei**	**Cytoplasm**	**Mitosis**	**Necrosis**	**LVI**	**Bi-/Multi-nucleation**	**Pleomo-** **rphism**	**Lymphocytic infiltrate**	**IHC**
1	+	-	Focal	Thick	10%	90%	Eosinophilic granular	1/10hpf	-	Present	-	-	-	TFE3 + Desmin+ in few cells MyoD1, S100, SOX10, HMB45, PAX8, EMA, CK, CK7, synaptophysin, chromogranin -ve
2	+	Focal	-	-	20%	80%	Clear	Absent	-	-	-	-	Minimal intratumoral	TFE3+ Desmin, MyoD1, EMA -ve
3	+	-	-	Thick	5%	95%	Clear	Absent	-	-	-	-	-	TFE3+ Desmin, MyoD1, EMA -ve
4	+	Focal	-	Thick	20%	80%	Eosinophilic granular	Absent	Present	Present	Present	Present	-	TFE3+ CD34, Synaptophysin, CK, MyoD1, SMA, S100 -ve
5	+	-	-	Thick	80%	20%	Eosinophilic granular	Absent	Present	-	-	-	-	TFE3 + CK5/6, CK7, p63, TTF1, CEA, PAX8, CK synaptophysin, PLAP, Oct3/4, EMA, myoglobin -ve
6	+	Focal	-	-	50%	50%	Eosinophilic granular	3/10hpf	-	Present	-	-	-	TFE3+ PAX8, CK, EMA, Vimentin, CK7, CD117, TTF1, synaptophysin, S100, CD10, HMB45, GFAP, desmin, CD34, Oct3/4 -ve
7	+	Focal	Focal	Thick Single cell infiltration	80%	20%	Eosinophilic granular	1/10hpf	-	Present	Occasional binucleated cell	-	Minimal peritumoral	TFE3+ Desmin, MyoD1, EMA, CK, PAX8 -ve
8	+	Focal	-	Thick	20%	80%	Eosinophilic granular	-	Present	-	-	-	Minimal intratumoral	TFE3 + CK, Vimentin, MyoD1, desmin, S100, synaptophysin -ve
9	+	-	-	-	40%	60%	Eosinophilic granular	-	Present	-	Present	-	-	TFE3+ CK, vimentin, S100, PLAP, EMA, CD34, Synpatophysin -ve
10	Focal	+	-	-	80%	20%	Eosinophilic granular	-	-	-	Present	-	-	TFE3+ CK, S100, PAX8, CD10, synaptophysin, chromogranin -ve
11	Focal	+	-	-	70%	30%	Eosinophilic granular	-	-	Present	Present	Present	-	TFE3 + Desmin, MyoD1, S100, HMB45, PAX8, EMA, AE1, CK7, synaptophysin, chromogranin -ve
12	+	-	-	Thick Single cell infiltration	10%	90%	Eosinophilic granular	-	-	Present	Occasional binucleated cell	Present	-	TFE3+ Desmin, MyoD1, S100, CK, Chromogranin, synaptophysin -ve
13	+	Focal	-	Thick	30%	70%	Eosinophilic granular	-	-	-	Present	-	-	TFE3+ Desmin, MyoD1, S100, CK, Chromogranin, synaptophysin -ve
14	Focal	+	-	Thick	80%	20%	Eosinophilic granular	1/10hpf	Present	Present	Present	-	-	TFE3+ Desmin, MyoD1, S100, CK, Chromogranin, synaptophysin -ve
15	+	Focal	-	-	30%	70%	Eosinophilic granular	-	-	-	-	-	-	TFE3+ S100 weak positive, Desmin, MyoD1, S100, CK, Chromogranin, synaptophysin -ve
16	Focal	+	Focal	Thick Single cell infiltration	30%	70%	Eosinophilic granular	2/10hpf	-	Present	Present	Present	-	TFE3+ SMA in few cells Vimentin, desmin, CD117, -ve
17	+	-	-	-	20%	80%	Eosinophilic granular	-	Present	Present	Present	-	-	TFE3+ CK, CK7, CD10, S100, Chromogranin, synaptophysin -ve
18	+	Focal	-	Thick	30%	70%	Eosinophilic granular	-	-	-	-	-	-	TFE3+ Vimentin, desmin, S100, myoglobin, CK, EMA, chromogranin, synaptophysin -ve
19	+	Focal	-	-	80%	20%	Eosinophilic granular	-	-	Present	Present	-	-	TFE3+ CK,S100,Desmin, HMB45 , EMA -ve
20	+	Focal	-	-	80%	20%	Eosinophilic granular	-	-	-	Present	-	-	TFE3+ CK,S100,Desmin, synaptophysin, chromogranin -ve
21	+	-	-	-	20%	80%	Eosinophilic granular	-	Present	Present	-	-	-	TFE3+ Desmin, synaptophysin, CK, -ve
22	+	-	-	-	30%	70%	Eosinophilic granular	-	-	Present	Present	-	-	TFE3+ Desmin: focal positive S100, CK, Chromogranin, synaptophysin, HMB45 -ve

**LVI:** Lymphovascular invasion, **IHC:** Immunohistochemistry, **CK:** Cytokeratin

All except one patient with localized disease of stage I-III were treated with surgical resection with clear margins, with no evidence of disease on follow-up.

Response was noted in 5 cases with tumor size <5 cm while only 3 cases out of 16 cases with a diameter >5 cm showed no evidence of disease on follow up. It was not affected by site in our study. A single patient of ASPS of the lung with stage I was treated with chemotherapy and radiotherapy with no response, rather progression of disease with metachronous metastasis to the liver and bone. All patients with disseminated disease i.e. stage IV were treated with anthracycline based chemotherapy and radiotherapy of 10 cycles of 30 gray. On regular follow-up, radiologically, no response but rather progression of disease was seen with increase in the size of the tumor at the primary site as well as an increase in the size of the metastasis. Out of 4 paediatric patients (age <17 years), a response was only noted in one case each of stage I and stage II cancers while another two of stage IV disease showed no response. No hospital death was reported in any patient. All were alive with disease in the limited period of follow-up ranging from 4 to 108 months.

## DISCUSSION

ASPSs are rare soft tissue tumors that constitute < 1% of all soft tissue sarcomas (1–3). The present study mirrors similar findings with only 0.4% the cases of ASPS out of all soft tissue sarcomas diagnosed over a period of 10 years. Studies have established that ASPS affects more commonly young adults; concordantly the age range in the present cohort was 2-47 years with four pediatric patients (1–16). The literature has a well documented female to male predominance before the age of 30 years, with a reversed ratio for older ages (1–7). Our study also corroborates these findings with the M:F ratio being 0.6:1 in patients less than 30 years while all three patients of age >30 years were male. However, Rekhi et. al. reported a male preponderance in their study (3). The prominent predilection for the extremities in our series is also well reflected in earlier studies (1–3,7,10). A rare site seen in the present series was primary pulmonary ASPS in a 25-year male without evidence of soft tissue tumor elsewhere at the time of initial diagnosis confirmed by the PET scan. To the best of our knowledge, only three cases of primary pulmonary ASPS have been reported in the English literature till date (8).

The clinical course in our series illustrates the high incidence of metastatic disease at the time of diagnosis with 50% of the cases. Many studies have reported metastatic disease at diagnosis in 55% to 65% of the patients (4,7). The most common metastatic site was the lung while brain metastasis was always a part of disseminated metastasis and never occurred in isolation, a phenomenon also observed by Portera et al. and Keyton et al (7,10). In our study, in two cases, metastases was detected prior to the finding of a primary, a phenomenon also encountered by other authors (2,12). One of our cases with the primary in the forearm also presented with metastasis in the nasal cavity which is not reported as the site of metastasis in any of the large series, though rare cases of primary sinonasal ASPS have been reported (1,3,7,9–12). Metastases to the lymph nodes are uncommon and were seen in only 2 cases in the present cohort. Portera et al. reported lymph node metastasis in a single patient only out of 70 cases (7). Our study had the first reported case of ASPS in patients with CMMRD (13). CMMRD is a childhood cancer predisposition syndrome caused by biallelic pathogenic variants in one of four mismatch repair (MMR) genes, i.e., *MLH1, MSH2, MSH6 *and *PMS2.* It is classically associated with hematological, brain, and intestinal malignancies but rare in sarcoma. Only 30 MMR deficient bone and soft tissue sarcomas including 3 ASPS were encountered in the literature (13,14). ASPS metastasis to the breast is considered extremely rare and is reported only in a handful of cases but was seen in one of our cases (15).

ASPS is known to have a very classical histomorphology showing very little variation from case to case and site to site. However, the diagnosis is challenging because of morphological overlap with other tumors, particularly on small biopsies and uncommon sites of occurrence or evaluation of metastatic site prior to identification of primary such as in biopsies from the posterior fossa, bone, or nasal cavity in the present series. Difficulties are further confounded by the occurrence of rare morphologic features particularly in biopsies such as solid pattern, clear cytoplasm, and unusual nuclear features.

With regard to the pattern, the tumor always had a lobular architecture with variably thick fibrous septae separating the lobules. We noted a significant preponderance of a ‘non-alveolar’ organoid growth pattern over the alveolar pattern, despite the name of the entity. This needs to be kept in mind, particularly when looking at a small biopsy.

Focal clear cytoplasm seen in two of our cases as a dominant feature raises the possibility for these cases to be confused with other clear cell tumors. The cells were also found to have a feathery kind of cytoplasm with condensation of the cytoplasm around the nucleus with pale cytoplasm at the periphery giving a lacy skirt kind of appearance.

Most of the studies in the literature including WHO 2013 and the latest WHO 2020 classification of tumors of soft tissue have emphasized vesicular nuclei with prominent eosinophilic nucleoli as a characteristic feature of ASPS but it was not the most prominent finding in the present series (1–12). The dominant nuclear feature (>50% of tumor nuclei) were bland nuclei with marked nuclear folding leading to concave, apple bite, and crenated nuclei without any nucleoli in nearly 68.2% of the cases and these nuclei were focal in the rest of the patients. These features were first observed by Fanburg-Smith et al. and Chatura et al. in lingual ASPS but it was an universal finding in the present series, independent of site (12,16). We also observed focal nuclear grooves in 3 cases which are not documented in the literature. Intranuclear inclusion was seen in one case and also observed in two cases by Rekhi et al (17). Awareness of these nuclear features is important and should not deviate one from the diagnosis of ASPS due to the absence of classical vesicular nuclei, particularly in small biopsies. The exact molecular pathogenetic relation between specific cellular-level structural features and cancer genes is not known. Nucleolar enlargement classically is associated with increased ribosome production, and production of new ribosomes appears essential for cell-cycle progression. Nuclear envelope irregularity may be the effect of downstream signaling pathway of the aberrant transcription factor ASPSCR1-TFE3 altering the structure of the nuclear membrane (18,19). Other rare features such as multinucleation and pleomorphism have been observed in other studies also but with no prognostic significance (2,3,12). Focal mucinous and cystic change reported in the literature was not seen in any of our cases.

Based on morphology, the differential diagnoses considered in the present study were paraganglioma, granular cell tumor, metastatic renal cell carcinoma, adrenocortical carcinoma, hepatocellular carcinoma, rhabdomyosarcoma, PEComa, and melanoma. Previously there was no specific marker for diagnosis of ASPS but the discovery of an unbalanced t(X::17) resulting in a fusion of the *ASPL* gene on chromosome 17 to the *TFE3* gene on chromosome X changed this scenario (1,5). Recently, novel *HNRNPH3-TFE3, DVL2-TFE3*, and *PRCC-TFE3* fusions have also been identified (6). Thus, immunodetection of the C terminus of the TFE3 protein in ASPS was considered a diagnostic landmark, but it should be interpreted carefully since the list of tumors with TFE3 immunopositivity is increasing. Cathepsin K is a cysteine protease abundantly expressed by osteoclasts and its expression is driven by microphthalmia transcription factor (MITF). *TFE3* also belongs to the same transcription factor subfamily as MITF. It is hypothesized that the TFE3 fusion proteins function like MITF in the neoplasms, and thus activate cathepsin K expression which can be detected by IHC (20).

*TFE3* rearrangements are not specific to ASPS but have also been identified in a subset of PEComa and a Mit Translocation renal cell carcinoma, both of which are morphological mimickers of ASPS. TFE3 immunoreactivity is not specific for TFE3 rearranged tumors, - Williams et al. have documented TFE3 positivity in four cases of granular cell tumors while Rekhi et al. observed TFE3 positivity in 28.5% of granular cell tumor (2,3). Cathepsin K immunoexpression is non-specific and has been reported in renal cell tumors, granular cell tumors, as well as numerous additional sarcomas including Kaposi sarcoma, liposarcoma, chondrosarcoma, undifferentiated pleomorphic sarcoma, and leiomyosarcoma (21). Granular cell tumors are diffusely immunopositive for S100, SOX10 and inhibin, which are negative in ASPS. There was focal weak S100 positivity in one of our tumors. Cytoplasmic granules can be also seen in granular cell tumor but PAS-positive diastase-resistant rod-like/rhomboid crystalline inclusions seen in 36.4% of the cases in the present series are specific for ASPS, and can be highlighted with MCT1 and CD147 immunostains while cytoplasmic granules in granular cell tumor are CD68 positive (1,3). Though TFE3 positivity have been reported in paraganglioma but immunopositivity for neuroendocrine markers, with S100 highlighting sustentacular cells, helps differentiate them from ASPS (3). PAX8, pan cytokeratin, CD10 negativity helps in ruling out renal cell carcinoma which is further substantiated by the absence of a renal mass on radiology. Negative immunostaining for vimentin and Melan-A ruled out an adrenocortical carcinoma. S100-P, HMB45, and Melan-A negativity in tumor cells ruled out a melanoma. Focal desmin positivity was seen in two of our cases but the lack of nuclear positivity for MyoD1 and myogenin ruled out a rhabdomyosarcoma. PEComa is differentiated from ASPS due to its reactivity for HMB45 but recently aberrant expression of HMB45 was also reported in ASPS, though both tumors are TFE3 rearranged, diagnosis of ASPS was favored based on presence of PAS-D needle crystals in ASPS (21). Translocation analysis can be performed, when necessary, and is the diagnostic ‘gold’ standard but one should be aware of other TFE3 rearranged tumors while interpreting the results (3,21).

None of our cases showed extensive mitosis or necrosis which are considered classical features of high-grade sarcoma. Despite thatbiological behavior of ASPS is aggressive, hence FNCLCC Histological Grading System isn’t used for them, all ASPS by definition are considered high grade (1).

The management of ASPS typically involves surgical resection for localized disease, which was performed in 8 cases and was curative. Anthracycline-based chemotherapy with or without radiotherapy was given for disseminated tumors with metastases in 10 cases and for localized disease in one case. It was largely ineffective with no response in any case and rather progression of disease was noted in all cases in present series. A search for novel therapies and their evaluation is being done in clinical trials. Molecular targeted treatment has been increasingly utilized. Vascular endothelial growth factor receptor-targeted TKIs such as pazopanib, crizotinib, sorafenib, anlotinib, sunitinib, and cedirranib and MET kinase inhibitors have been explored in clinical trials for metastatic disease with promising results (5,22). We argue against the future of immunotherapy in ASPS since a very focal intratumoral inflammatory host response was seen in only two cases and only one case showed minimal lymphocytic response at the tumor edge.

## CONCLUSION

ASPS has a morphological and immunohistochemical overlap with many mesenchymal and non-mesenchymal tumors. Diffuse strong nuclear TFE3 positivity is sensitive for ASPS in an appropriate clinicoradiological context. Awareness of TFE3 positivity in other tumors is vital. It is imperative to employ a panel of markers in order to identify an alveolar soft part sarcoma from its differential diagnoses. Due to the high propensity for early metastasis even at the time of presentation in ASPS, complete metastatic workup and long term follow up is recommended. ASPS is associated with slow progression and resistance to conventional cytotoxic chemotherapy.

## Ethics Approval and Consent to Participate

The study has been approved by the institute research committee of GCRI assuring legal and ethical criteria fulfilment in the study with review number IRC/2022/P-79.

## Funding

Authors received no financial support for the research, authorship and/or publication of this manuscript

## Conflict of Interest

The authors declare that they have no competing interests.

## Availability of Data and Material

Available on request from the corresponding author.
